# An Operating Stiffness Controller for the Medical Continuum Robot Based on Impedance Control

**DOI:** 10.34133/cbsystems.0110

**Published:** 2024-05-08

**Authors:** Jinyu Duan, Kai Zhang, Kun Qian, Jianxiong Hao, Zhiqiang Zhang, Chaoyang Shi

**Affiliations:** ^1^ Key Laboratory of Mechanism Theory and Equipment Design of Ministry of Education, School of Mechanical Engineering, Tianjin University, Tianjin 300072, China.; ^2^ School of Engineering and Physical Sciences, Heriot-Watt University, Edinburgh EH14 4AS, UK.; ^3^ School of Electronic and Electrical Engineering, University of Leeds, Leeds LS2 9JT, UK.

## Abstract

Continuum manipulators can conform to curvilinear paths and manipulate objects in complex environments, which makes it emerging to be applied in minimally invasive surgery (MIS). However, different and controllable operating stiffness of the continuum manipulator is required during different stages of surgery to achieve safe access or stable and precise operation. This work proposes an operating stiffness controller (OSC) for the typical tendon-driven continuum manipulator based on the variable impedance control method with Lagrangian dynamic modeling. This controller can adjust the operating stiffness by modifying the driving forces along the driving tendons of the continuum manipulator without changing its material or structure. The proposed OSC converts the damping and stiffness matrices of the impedance control into variable parameters. This merit allows it to dynamically adjust the operating stiffness of the continuum manipulator according to the desired constant or time-varying stiffness. Furthermore, the OSC stability can be proven based on a Lyapunov function, and its stiffness control performances have been analyzed and evaluated in both simulations and experiments. The OSC controller generated average relevant error values of 7.82% and 3.09% for the operating stiffness control experiments with constant and time-varying desired stiffness, respectively. These experimental results indicate that the OSC has high accuracy, stability, and strong robustness in the operating stiffness control tasks.

## Introduction

**Fig. 1.**
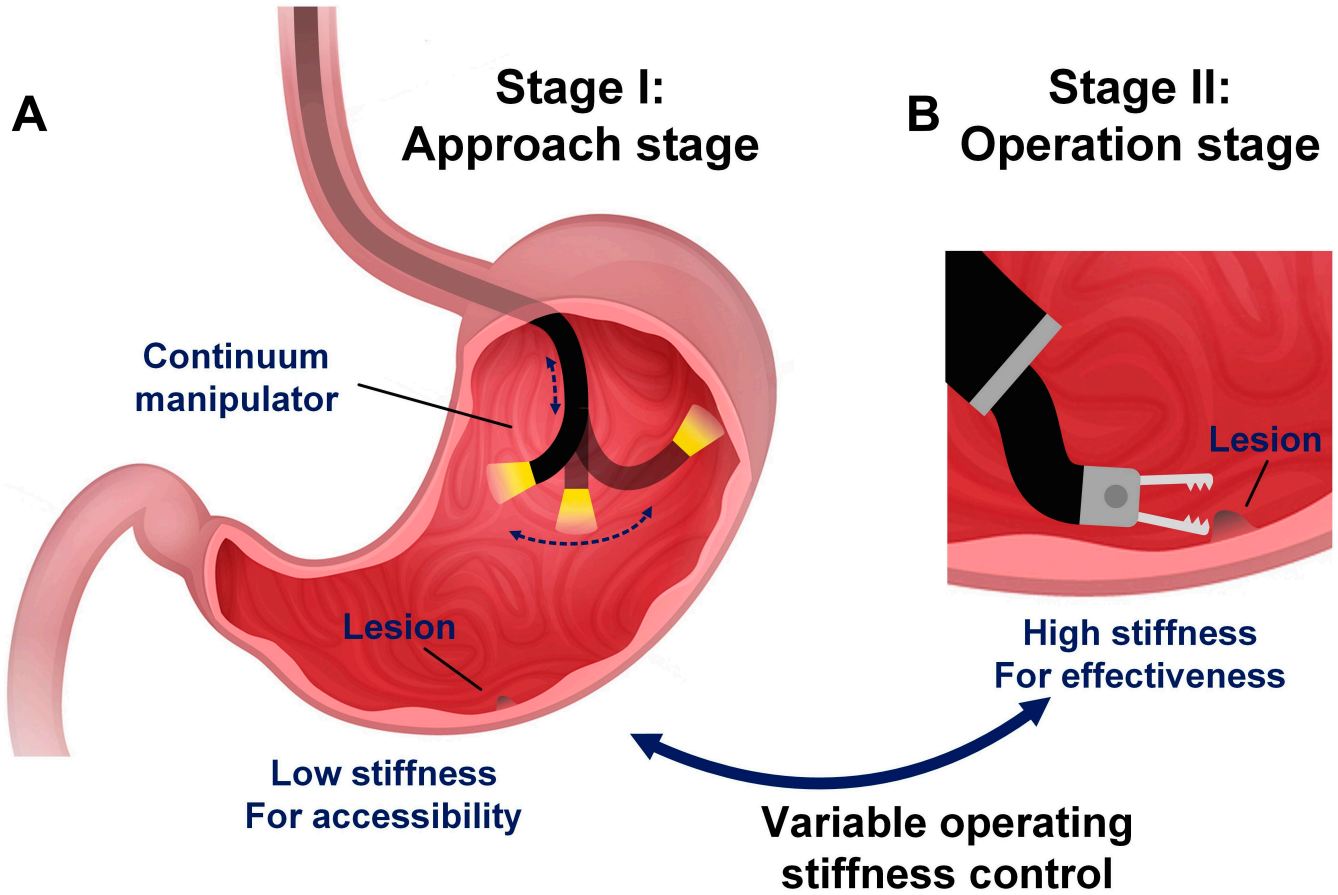
Different operating stiffness levels are required at various stages of surgery to achieve different objectives. The transition of operating stiffness between stages can be facilitated by the stiffness control. (A) Low stiffness is needed for accessibility in the approach stage. (B) High stiffness is needed for effectiveness in the operation stage.

Robotic-assisted flexible endoscopy is an advanced no-visible-scar surgery technique for minimally invasive surgery (MIS) and has been increasingly utilized for diagnosis and treatment. The surgical outcomes have proven the potential and reliability of such advanced endoscopic techniques in performing internal screening and operations through natural orifices [1–4]. The continuum manipulators have exhibited great potential and promising applicability for these robotic systems due to their slender and compact design, excellent curvilinearity, inherent structural compliance, and flexible access and dexterity in narrow and tortuous pathways [5–9]. Unlike traditional rigid robots, the inherent flexibility of continuum robots enhances their safety during navigation access to the surgical sites [10–14]. However, in actual surgical scenarios, continuum manipulators not only need excellent accessibility to facilitate flexible and safe access to the instruments but also require sufficient effectiveness to contribute to stable and precise operations of the instrument, which demands greater stiffness [15], as shown in Fig. 1. As a result, realizing the stiffness change of a continuum manipulator during MIS is demanding and challenging [16]. For those continuum manipulators with low inherent stiffness, several implementations based on variable stiffness materials or special mechanical designs have been developed to enhance and control their stiffness [17]. For those situations when the inherent stiffness is sufficient, control algorithms that modify the kinematic inputs to achieve the desired contact stiffness have been developed [18].

**Table 1.** Parameters of simulations

Parameters of simulations	Description	Value
***α***	The stability condition constant	1
***M***_**d**_	The desired inertia matrix	$\left[\begin{array}{cc} 1 & 0\\ 0 & 1 \end{array}\right]\mathrm{kg}\;$
***D***_**d**_	The desired damping matrix	300***M***_d_
***K***_**d**_	The desired operating stiffness matrix	$\left[\begin{array}{cc} k_{1} & 0\\ 0 & k_{2} \end{array}\right]N\cdot m^{-1}$
***F***_**ext**_	The external force	$\left[\begin{array}{c} -1\\ -1 \end{array}\right]N\;$

In the field of materials engineering, smart fluids [magnetorheological (MR) or electrorheological (ER) fluids] [19,20], low melting point alloys (LMPAs) [21–24], thermoplastic polymer [25,26], or shape memory materials [27–29] are widely utilized for stiffness adjustment by changing the intrinsic material properties with external stimulation. However, since these external stimulations are typically performed based on heat conduction or electromagnetic fields, the phase transition process of these materials is commonly time-consuming [30]. Moreover, applying these external stimulations requires additional energy exchangers, resulting in larger joint diameters, poor biocompatibility, and high leakage risks. An alternative approach for stiffness adjustment includes mechanical designs like unit-locking mechanisms [31,32] and jamming-based methods [33–37]. To adjust manipulator stiffness, unit-locking mechanisms reorganize mechanical engagement through gears, racks, etc. [10]. However, complex structures pose challenges in diameter/size reduction, and discontinuous bending motion arising from the discrete configuration limits their utilization in endoscopic surgery.

The other main research stream employs control algorithms to modify the operating stiffness of the continuum manipulator without altering the material and mechanical structure of continuum manipulators. These methods encompass control algorithms based on static modeling or dynamic modeling. The static modeling-based control algorithms provided an approach for stiffness control on any continuum manipulator [38]. However, this algorithm is limited to regulating constant desired stiffness and lacks mathematical proof of controller stability. Additionally, static modeling is deficient in terms of dynamic performance and robustness compared with dynamic modeling. The Lagrangian dynamic modeling-based approaches [39,40] have addressed the issues of time-varying desired stiffness and stability proof of the controller to some extent. However, these methods still lack quantitative analysis and evaluation of the continuum manipulators’ actual operating stiffness. In conclusion, only a few studies utilize control algorithms for stiffness control in continuum manipulators, and the existing works based on control algorithms lack quantitative studies on operating stiffness control.

To address the above issues, a Lagrangian dynamic-based operating stiffness controller (OSC) is introduced in this work, enabling continuous and quantitative control of operating stiffness for tendon-driven continuum manipulators. Compared with controllers based on static models, the designed controller exhibits improved dynamic and interactive performance. This controller consists of a variable impedance control module followed by a feedback linearization module and supports adjusting the operating stiffness via the forces in the driving tendons. The variable impedance control module [41–43] multiplies the continuum manipulator’s distal movement state by given impedance parameters, resulting in the continuum manipulator’s distal contact force. This module also enables the continuum manipulator’s distal tip to exhibit the desired stiffness characteristics. The feedback linearization module is based on the Lagrangian dynamics model. This module converts the distal movement state into the sum of the required driving force and distal contact force. By taking the difference between the outputs of these two modules, the proposed OSC outputs the required driving force while eliminating the contact force term. This feature allows the OSC to achieve stiffness control without relying on distal force sensors, aligning with the compact distal tip structure of continuum manipulators. Moreover, the stability of the OSC is proven based on an energy-weighted Lyapunov function. Furthermore, based on the definition of the continuum manipulator’s distal tip stiffness [44,45], this work defines the operating stiffness of continuum manipulators, which is more consistent with the application scenario of surgery. Based on the Lagrangian dynamics model, this definition is decomposed into active and passive adjustment terms. This result demonstrates that operating stiffness can be actively adjusted through driving forces, laying the foundation for the design of OSC. Finally, quantitative analysis and evaluation of the proposed controller’s performance are conducted in experiments and simulations based on the defined operating stiffness. This quantitative analysis is lacking in existing works [39,40]. The simulation and experimental results demonstrate the OSC’s continuous and effective adjustment of the continuum manipulator’s operating stiffness according to different desired operating stiffness settings.

## Materials and Methods

### Definition of operating stiffness of the continuum manipulator

In this work, the operating stiffness ***K*** ∈ *ℝ*^2×2^ of the continuum manipulator is defined as the partial derivative of the contact force ***F***_ext_ ∈ *ℝ*^2×1^ to the displacement ***x*** ∈ *ℝ*^2×1^ in the workspace, as expressed below:

$$K=\frac{\partial F_{\mathrm{ext}}}{\partial x}$$ (1)

However, it is the driving force ***F***_q_ ∈ *ℝ*^2×1^ along the tendon that is the output of the force controller for a tendon-driven continuum manipulator. Therefore, the operating stiffness of the continuum manipulator needs to be expressed in terms of the driving force. This necessitates finding the mapping relationship between the continuum manipulator’s contact and driving forces. This mapping relationship can be established by the principle of virtual work, as shown in Eq. 2:

$$F_{q}^{T}\Delta q={F_{\mathrm{ext}}}^{T}\Delta x$$ (2)

where ***F***_q_ and Δ***q*** ∈ *ℝ*^2×1^ respectively represent the driving force and the infinitesimal displacement of the driving tendon in the configuration space, and ***F***_ext_ and Δ***x*** denote the contact force and the infinitesimal displacement of the continuum manipulator’s distal tip in the workspace, respectively.

Based on our previous work [46], the infinitesimal distal tip displacement of a continuum manipulator is related to its infinitesimal driving tendon displacement Δ***q***, as shown in Eq. 3:

Δx=JΔq (3)

where ***J*** ∈ *ℝ*^2×2^ denotes the Jacobian matrix of the continuum manipulator’s kinematics model.

By combining Eqs. 2 and 3, the relationship between the driving force and the contact force can be derived as follows:

$$F_{\mathrm{ext}}=J^{-T}F_{q}$$ (4)

By substituting Eq. 4 into Eq. 1, the operating stiffness of the continuum manipulator defined in Eq. 1 can be indicated by the driving force as follows:

$$K=\frac{\partial F_{\mathrm{ext}}}{\partial x}=\frac{\partial \left(J^{-T}F_{q}\right)}{\partial x}=\frac{\partial J^{-T}}{\partial x}F_{q}+J^{-T}\frac{\partial F_{q}}{\partial x}$$ (5)

By applying the chain rule, the last term of Eq. 5 can be written as follows:

$$J^{-T}\frac{\partial F_{q}}{\partial x}=J^{-T}\frac{\partial F_{q}}{\partial q}\frac{\partial q}{\partial x}=J^{-T}k_{q}J^{-1}$$ (6)

where ***k***_q_ ∈ *ℝ*^2×2^ denotes the tensile stiffness of the driving tendon in the tendon-driven continuum manipulator.

By substituting Eq. 6 into Eq. 5, the final form of the operating stiffness can be obtained as Eq. 7:

$$K=\frac{\partial J^{-T}}{\partial x}F_{q}+J^{-T}k_{q}J^{-1}$$ (7)

Each term of Eq. 7 is equivalent to the corresponding term of Eq. 5. From Eq. 7, it can be inferred that the operating stiffness of the continuum manipulator consists of two components: the passive component ***J***^−*T*^***k***_q_***J***^−1^ (***J***^−*T*^ · *∂****F***_q_/*∂****x*** in Eq. 5) and the active component (*∂****J***^−*T*^/*∂****x***)***F***_q_. The passive component is determined by the tensile stiffness of the drive tendons and the configuration state and cannot be altered arbitrarily during the motion process. However, the value of the active component can be actively and extensively adjusted by varying the driving forces of the driving tendons. Therefore, the operating stiffness of a tendon-driven continuum manipulator can be actively controlled by adjusting the driving forces in the driving tendons. This provides a theoretical basis for the design of the OSC in the subsequent sections.

### Design of OSC for the continuum manipulator based on variable impedance control method

**Fig. 2.**
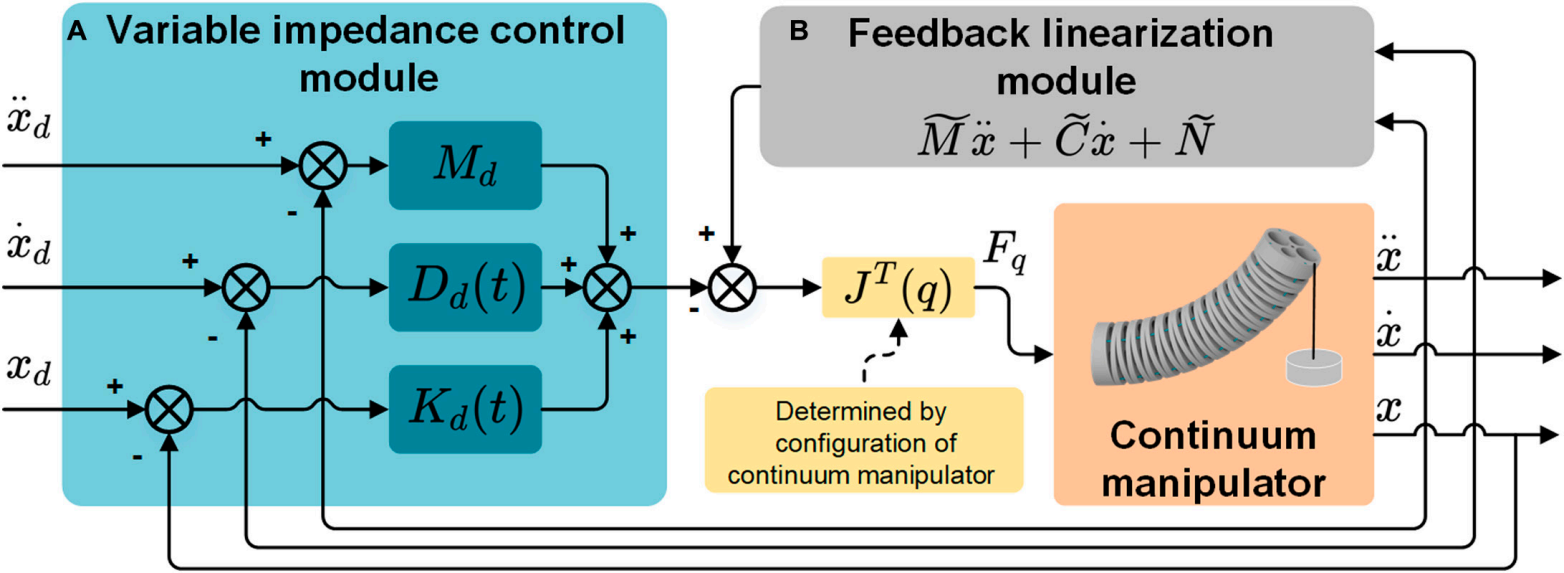
The proposed OSC. (A) The variable impedance control module multiplies the continuum manipulator’s distal movement state by given impedance parameters, resulting in the continuum manipulator’s distal contact force. (B) The feedback linearization module converts the distal movement state into the sum of the required driving force and distal contact force.

An OSC based on a variable impedance control method is proposed to adjust the operating stiffness of the continuum manipulator according to the constant or time-varying desired operating stiffness. The schematic overview of the controller is illustrated in Fig. 2. This controller quantitatively alters the operating stiffness by modifying the driving force of the continuum manipulator rather than changing the material or structure of the continuum manipulator. The specific expression of the variable impedance controller is as follows:

$$M_{d}\ddot{\tilde{x}}+D_{d}\dot{\tilde{x}}+K_{d}\left(t\right)\tilde{x}=F_{\mathrm{ext}}$$ (8)

where ***M***_d_ ∈ *ℝ*^2×2^ and ***D***_d_ ∈ *ℝ*^2×2^ respectively represent the desired inertia and damping matrix for the control system, and ***K***_d_(*t*) ∈ *ℝ*^2×2^ denotes the desired time-varying stiffness matrix for the control system. $\tilde{x}\in {\mathbb{R}}^{2\times 1}$ represents the distal tip position error of the continuum manipulator, and ***F***_ext_ ∈ *ℝ*^2×1^ denotes the contact force acting on the continuum manipulator.

**Table 2.** Parameters of the continuum manipulator in simulations

Description	Value
The length of the continuum manipulator	0.2 m
The radius of the continuum manipulator	0.01 m
The mass of the continuum manipulator	0.02 kg
The Young’s modulus	60 GPa
Moments of inertia	5.153 × 10^−13^ m^4^

The stiffness matrix ***K***_d_(*t*) in Eq. 8 can be determined by the actual surgical requirements. The damping matrix ***D***_d_ is set to be a multiple of the inertia matrix ***M***_d_ to ensure the stability of the continuum manipulator. Moreover, according to the stability conditions under dynamic decoupling in [47], ***M***_d_ should be a constant, symmetric, and positive definite matrix. ***D***_d_ and ***K***_d_(*t*) should be symmetric, positive definite, and continuously differentiable varying damping and stiffness matrices.

To obtain the control law of the proposed OSC, it is necessary to introduce the dynamics model of the continuum manipulator. Based on some classical dynamic modeling efforts [48], the dynamics model of the continuum manipulator subjected to external forces is given by Eq. 9:

$$M\left(q\right)\ddot{q}+C\left(q,\overset{\cdot }{q}\right)\overset{\cdot }{q}+N\left(q\right)=F_{q}+J^{T}F_{\mathrm{ext}}$$ (9)

However, Eq. 9 is the dynamics model of the continuum manipulator related to the displacements of the driving tendons, while the desired dynamic characteristics of the control system are related to the displacement of the continuum manipulator’s distal tip. Therefore, it is necessary to transform Eq. 9 into the dynamics model related to the distal tip of the continuum manipulator. According to the differential kinematics of the continuum manipulator, the relationships between the velocity and acceleration of a continuum manipulator’s distal tip and tendon displacement are shown in Eqs. 10 and 11:

$$\overset{\cdot }{x}=J\overset{\cdot }{q}$$ (10)

$$\ddot{x}=\overset{\cdot }{J}\overset{\cdot }{q}+J\ddot{q}$$ (11)

where $\overset{\cdot }{x}$ and $\overset{\cdot }{q}$ respectively denote the velocity of the continuum manipulator’s distal tip and tendon displacement, and $\ddot{x}$ and $\ddot{q}$ represent the acceleration of the continuum manipulator’s distal tip and tendon displacement, respectively.

By substituting Eqs. 10 and 11 into Eq. 9, the dynamics model of the continuum manipulator related to the distal tip displacement can be given as Eq. 12:

$$M\left(q\right)J^{-1}\left(\ddot{x}-\overset{\cdot }{x}\right)+C\left(q,\overset{\cdot }{q}\right)J^{-1}\overset{\cdot }{x}+N\left(q\right)=F_{q}+J^{T}F_{\mathrm{ext}}$$ (12)

After simplifying and rearranging Eq. 12, the following expression can be obtained:

M~x¨+C~x·+N~=J−TFq+FextM~=J−TMqJ−1N~=J−TNqC~=J−TCq,q·J−1−J−TMqJ−1J·J−1 (13)

By combining Eqs. 8 and 13, the desired control law of the proposed OSC can be derived as follows:

$$F_{q}=J^{T}\left(q\right)\left(\tilde{M}\ddot{x}+\tilde{C}\overset{\cdot }{x}+\tilde{N}-M_{d}\ddot{\tilde{x}}-D_{d}\overset{\cdot }{\tilde{x}}-K_{d}\left(t\right)\tilde{x}\right)$$ (14)

Under this control law, the continuum manipulator exhibits the desired dynamic characteristics, as stated in Eq. 8. The first three terms and the last three terms in Eq. 14 correspond to the feedback linearization module and the variable impedance control module in Fig. 2. The feedback linearization module here eliminated the contact force term ***F***_ext_. This feature helps the OSC to achieve stiffness control without distal force sensors, aligning with the compact distal tip structure of continuum manipulators. In the actual medical scenario, the desired operating stiffness ***K***_d_(*t*) can be manually given in specific surgical stages. At the same time, the distal tip position, velocity, and acceleration in Eq. 14 can be obtained by intraoperative imaging methods such as endoscopes in practical applications. When external forces exist, the continuum manipulator will track the desired trajectory with stable position error and the desired operating stiffness. Moreover, this controller adjusts the operating stiffness by modifying the driving force of the continuum manipulator, instead of changing the material or structure of the continuum manipulator. This control law also enables the quantitative control of the continuum manipulator’s operating stiffness.

**Table 3.** The experimental results of the OSC in the experiment with constant desired operating stiffness at the fixed distal tip position

The desired operating stiffness (N/m)	The average actual operating stiffness (N/m)	Relevant error of operating stiffness	Standard deviation of operating stiffness (N/m)
10	7.67	23.33%	23.81
15	13.86	7.60%	13.20
20	18.84	5.80%	9.72
25	24.62	1.52%	7.44
30	29.75	0.83%	6.16
Mean value on five experiments	/	7.82%	12.07

### Stability proof of OSC for the continuum manipulator

A weighted energy function based on the velocity error and position error is chosen to construct a stability condition related to the stiffness and damping matrices of the continuum robot and independent of other state variables. The corresponding Lyapunov function is shown as follows:

$$\left(\dot{\tilde{x}},\tilde{x},t\right)=\frac{1}{2}{\left(\dot{\tilde{x}}+\alpha \tilde{x}\right)}^{T}M_{d}\left(\dot{\tilde{x}}+\alpha \tilde{x}\right)+\frac{1}{2}{\tilde{x}}^{T}\beta \left(t\right)\tilde{x}$$ (15)

where *α* denotes the stability condition constant and *α* > 0. ***β***(*t*) is a positive semidefinite matrix. The derivative of the Lyapunov function is given by:

$$\overset{\cdot }{V}\left(\overset{\cdot }{\tilde{x}},\tilde{x},t\right)={\left(\overset{\cdot }{\tilde{x}}+\alpha \tilde{x}\right)}^{T}M_{d}\left(\ddot{\tilde{x}}+\alpha \overset{\cdot }{\tilde{x}}\right)+{\tilde{x}}^{T}\beta \left(t\right)\overset{\cdot }{\tilde{x}}+\frac{1}{2}{\tilde{x}}^{T}\overset{\cdot }{\beta }\left(t\right)\tilde{x}$$ (16)

Let the contact force ***F***_ext_ in Eq. 8 be zero, then the relationship between ***M***_d_, ***D***_d_, and ***K***_d_(*t*) can be given as:

$$M_{d}\ddot{\tilde{x}}=-D_{d}\overset{\cdot }{\tilde{x}}-K_{d}\left(t\right)\tilde{x}$$ (17)

By substituting Eq. 17 into Eq. 16, the derivative of the Lyapunov function can be rewritten as:

$$\begin{array}{c} \dot{V}\left(\dot{\tilde{x}},\tilde{x},t\right)={\dot{\tilde{x}}}^{T}\left(\alpha M_{d}-D_{d}\right)\dot{\tilde{x}}+{\tilde{x}}^{T}\left(-\alpha K_{d}\left(t\right)+\frac{1}{2}\dot{\beta }\left(t\right)\right)\\ \; \end{array}$$ (18)

*** β***(*t*) can be defined as the following positive semidefinite matrix:

$$\beta \left(t\right)=K_{d}\left(t\right)+\alpha D_{d}-\alpha^{2}M_{d}$$ (19)

*Proof:*

Since ***K***_d_(*t*) is a positive semidefinite matrix, *α* > 0:

$$\beta \left(t\right)=K_{d}\left(t\right)+\alpha \left(D_{d}-\alpha M_{d}\right)$$ (20)

then ***β***(*t*) is a positive semidefinite matrix if *α****M***_d_ − ***D***_d_ < 0.∎

By substituting Eq. 19 into Eq. 18, the derivative of the Lyapunov function can be rewritten as:

$$\dot{V}\left(\dot{\tilde{x}},\tilde{x},t\right)={\tilde{x}}^{T}\left(-\alpha K_{d}\left(t\right)+\frac{1}{2}{\dot{K}}_{d}\left(t\right)\right)\tilde{x}+{\dot{\tilde{x}}}^{T}\left(\alpha M_{d}-D_{d}\right)\dot{\tilde{x}}$$ (21)

According to Eq. 21, the proposed OSC needs to satisfy the following conditions in Eq. 22 to ensure the derivative of the Lyapunov function $\overset{\cdot }{V}\left(\overset{\cdot }{\tilde{x}},\tilde{x},t\right)<0$:

$$\left\{\begin{array}{c} \alpha >0\\ \alpha M_{d}-D_{d}<0\\ {\dot{K}}_{d}\left(t\right)-2\alpha K_{d}\left(t\right)<0. \end{array}\right.$$ (22)

Furthermore, the control system is asymptotically stable because the Lyapunov function is always greater than zero.

## Experiments and Results

**Fig. 3.**
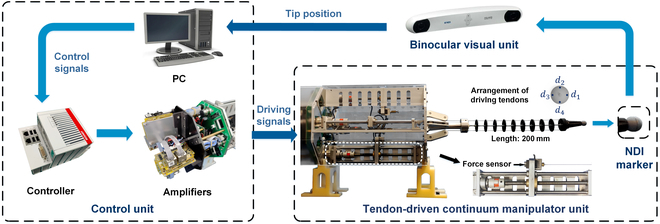
Hardware configuration of the continuum robot system. Note: *d_i_* demotes the *i*th driving tendon of the continuum manipulator.

The overview of the continuum robot system has been illustrated in Fig. 3. The experimental validation of the proposed approach is conducted utilizing a spacing disk-type continuum manipulator. This continuum manipulator is operated via four driving tendons that enable bending motions in pitch and yaw. The displacements of these four tendons are obtained by converting the rotary motion of the DC motors (Maxon, DC16, Switzerland) into linear motion utilizing four linear modules. Amplifiers (MotionG, UF-48V10AEDR, China) are utilized to drive and power these modules and simultaneously regulate the driving forces on the tendons. These driving forces are measured by force sensors (JinNuo, BSQ-2, China) integrated into the linear modules. The binocular visual unit (NDI Polaris, Ontario, Canada) measures the distal tip position of the continuum manipulators with the distally attached marker. The measured position data are sent to the host computer (Core i7 processor @ 2.80 GHz 16-GB RAM) for position feedback with a sampling rate of 60 Hz through a USB cable. The central motion controller (CX-5140, Beckhoff, Germany) receives the control signals from the host computer and sends these commands to the amplifiers (MotionG, UF-48V10AEDR, China) to drive these DC motors.

**Table 4.** The experimental results of the OSC in the experiment with constant desired operating stiffness on the circle trajectory

The desired operating stiffness (N/m)	The average actual operating stiffness (N/m)	Relevant error of operating stiffness	Max error of operating stiffness
35	33.30	4.86%	44.23%
70	69.08	1.31%	38.33%
Mean value on two experiments	\	3.09%	41.33%

### Simulations of the presented OSC for the continuum manipulator

To validate that the proposed OSC controller can effectively adjust the operating stiffness of the continuum manipulator, the following simulations with different desired stiffness conditions were conducted. In each simulation, the continuum manipulator was initially positioned at a fixed location and maintained stationary. An external force was applied at a designated moment, and the deviation of the continuum manipulator’s distal tip position from the desired position was observed. The parameters of the continuum manipulator and parameters of each simulation have been emulated in Tables 1 and 2. The current operating stiffness of the continuum manipulator was calculated according to Eq. 1.

#### Simulation A: Simulation with constant desired operating stiffness

**Fig. 4.**
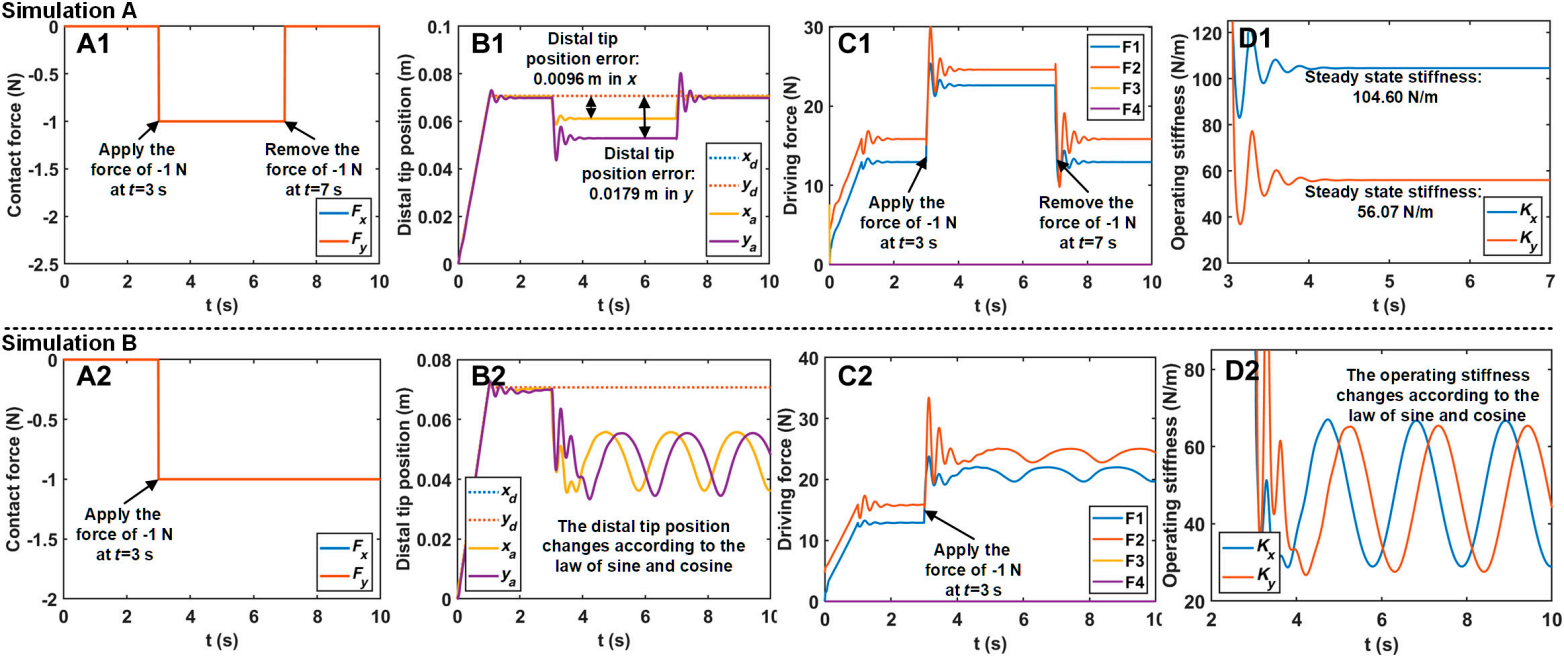
The simulation results of OSC for the continuum manipulator with constant desired operating stiffness and time-varying desired operating stiffness. (A1 and A2) Magnitude and duration of the contact force in the simulation. (B1 and B2) Distal tip position in both *x* and *y* directions of the continuum manipulator in the simulation. (C1 and C2) Driving force on every driving tendon in the simulation. (D1 and D2) Operating stiffness of the continuum manipulator during the application of the contact force in the simulation. Note: F1 to F4 in (C1) and (C2) represent the driving force on the first to fourth driving tendon, respectively.

By setting different desired stiffness values in the *x* and *y* directions (*k*_1_ and *k*_2_ in this simulation were respectively set as 100 and 50), this simulation enables the reflection of the effectiveness of OSC by observing distinct position errors in both directions. The simulation results are calculated, as illustrated in Fig. 4. During the simulation, a contact force was applied at *t* = 3 s and removed at *t* = 7 s (Fig. 4A1). It is observed that with increased stiffness, the displacement of the distal tip obviously decreases (Fig. 4B1). It is important to note that in Fig. 4C1, the higher driving force in the *y* direction is attributed to the need for the additional driving force to counterbalance the influence of gravity in that direction. Further calculations of the operating stiffness of the continuum manipulator reveal that during the application of external force, the operating stiffness respectively stabilizes at 104.60 N/m and 56.07 N/m in the *x* and *y* directions, closely matching the desired operating stiffness values (100 N/m and 50 N/m) (Fig. 4D1). This result suggests that the OSC has the capability to efficiently modify and maintain the operating stiffness of the continuum manipulator.

**Fig. 5.**
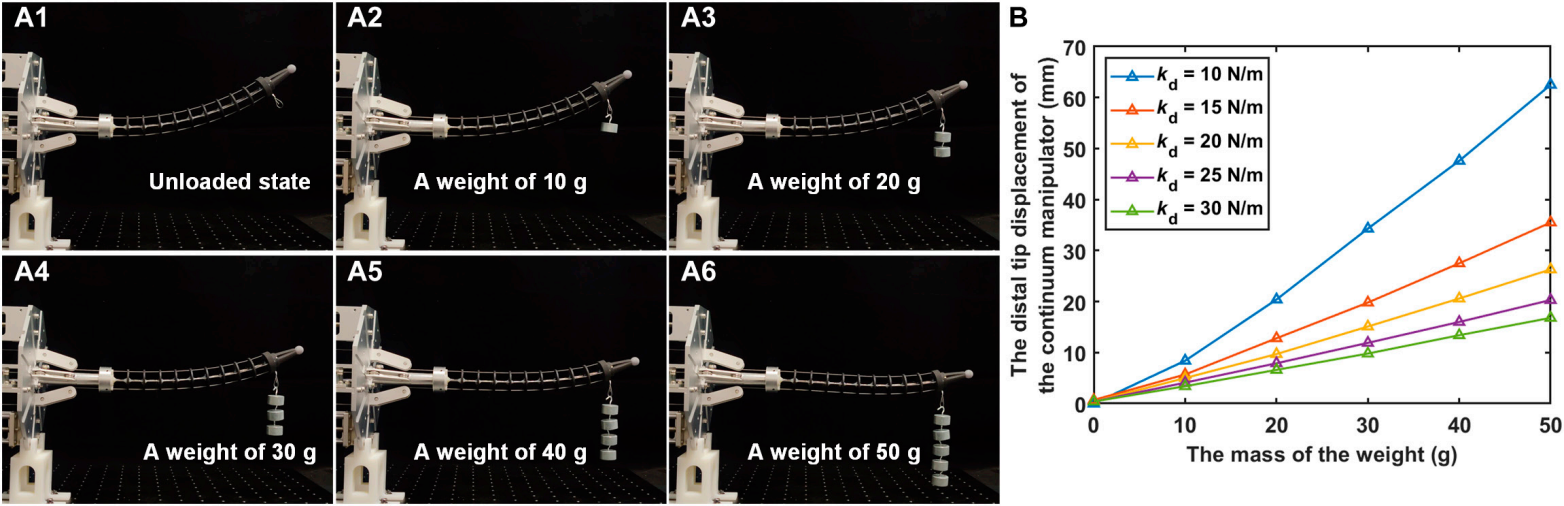
The distal tip displacement of the continuum manipulator and the operating stiffness curve in the operating stiffness control experiment with constant desired operating stiffness at the fixed desired distal tip position. (A1 to A6) Physical illustrations of the distal tip of the continuum manipulator under different load conditions. (B) Relationship between the displacement of the continuum manipulator’s distal tip and the mass of the load weight under different desired operating stiffness conditions.

#### Simulation B: Simulation with time-varying desired operating stiffness

Different from simulation A, the desired operating stiffness values in the *x* and *y* directions in this simulation have been set as sinusoidal pattern time-varying functions. *k*_1_ and *k*_2_ in this simulation were respectively set as 40 + 20 sin (3*t*) and 40 + 20 sin (3*t* − π/2). The objective is to verify whether the OSC can efficaciously enable the continuum manipulator to track the desired time-varying operating stiffness values.

The simulation results are calculated as shown in Fig. 4. During the simulation, a contact force was applied at *t* = 3 s until the end of the simulation (Fig. 4A2). Under the influence of the constant contact force, the distal tip of the continuum manipulator exhibits sinusoidal-like oscillations (Fig. 4B2). This behavior is attributed to the fact that the operating stiffness of the continuum manipulator varies according to the desired sinusoidal pattern. The discrepancy in the magnitudes of driving forces in the *x* and *y* directions is consistent with the factors observed in simulation A (Fig. 4C2). Additional computations of the operating stiffness of the continuum manipulator reveal that the operating stiffness follows the prescribed sinusoidal pattern (Fig. 4D2). These results demonstrate the OSC’s efficacy in adapting the continuum manipulator’s operating stiffness according to the specified time-varying operating stiffness.

### Experiments of the presented OSC with constant desired operating stiffness for the continuum manipulator

#### Experiments with constant desired operating stiffness at the fixed distal tip position

The following experiments were performed to validate the ability of the proposed OSC to effectively adjust the operating stiffness of a continuum manipulator in a static state according to the desired operating stiffness. *α*, ***M***_d_, and ***D***_d_ remain the same as those in simulations. Initially, the distal tip of the continuum manipulator was moved to a fixed position and held static, and the distal tip position was recorded as the reference distal tip position. Subsequently, a standard weight of 10 g was added one by one at a time. After each addition, the displacement in the *y* direction between the current distal tip position and the reference distal tip position was recorded. A maximum of five weights were suspended in each experiment. Five sets of experiments were conducted based on different desired operating stiffness values (from 10 N/m to 30 N/m with an interval of 5 N/m). The experimental procedure is illustrated in Fig. 5A1 to A6.

The experimental results were calculated and compared, as shown in Fig. 5B and Table 3. In these experiments, the OSC generated an average relative error of 7.82% and a standard deviation of 12.07 N/m between the actual operating stiffness and the desired operating stiffness of the continuum manipulator. Observing the results across multiple trials, it was found that the relative error and the standard deviation in the operating stiffness of the continuum manipulator decreased with an increase in the desired operating stiffness (respectively from 23.33% to 0.83% and from 23.81 N/m to 6.16 N/m). This result is attributed to the fact that reducing the desired operating stiffness makes the passive compliance of the continuum manipulator more pronounced, thus making it more susceptible to disturbances under loaded conditions. These experimental results indicate the proposed OSC’s capability of precisely adjusting the operating stiffness of a continuum manipulator in a static state for the desired operating stiffness. What is more, the ability of the continuum manipulator to switch operating stiffness demonstrated in this experiment is consistent with the requirements of the approaching stage in actual surgeries. These results also reflect that the proposed OSC can be efficiently applied in actual surgical scenarios.

#### Experiments with constant desired operating stiffness on the circular trajectory

To confirm the efficacy of the proposed OSC in adapting the operating stiffness of the continuum manipulator in motion as the desired operating stiffness, experiments were conducted under different desired stiffness settings. *α*, ***M***_d_, and ***D***_d_ remain the same as those in simulations. The continuum manipulator was tasked with tracking a circular trajectory, as illustrated in Eq. 23, at the specified desired operating stiffness. A 50-g weight was added at the beginning, and the movement of the continuum manipulator lasted for two loops. The dynamic characteristics of the continuum manipulator were observed during the experiments. Two sets of experiments were conducted, and the desired stiffness settings for each experiment were 35 N/m and 70 N/m, respectively.

$$x^{2}+y^{2}=75^{2}$$ (23)

**Fig. 6.**
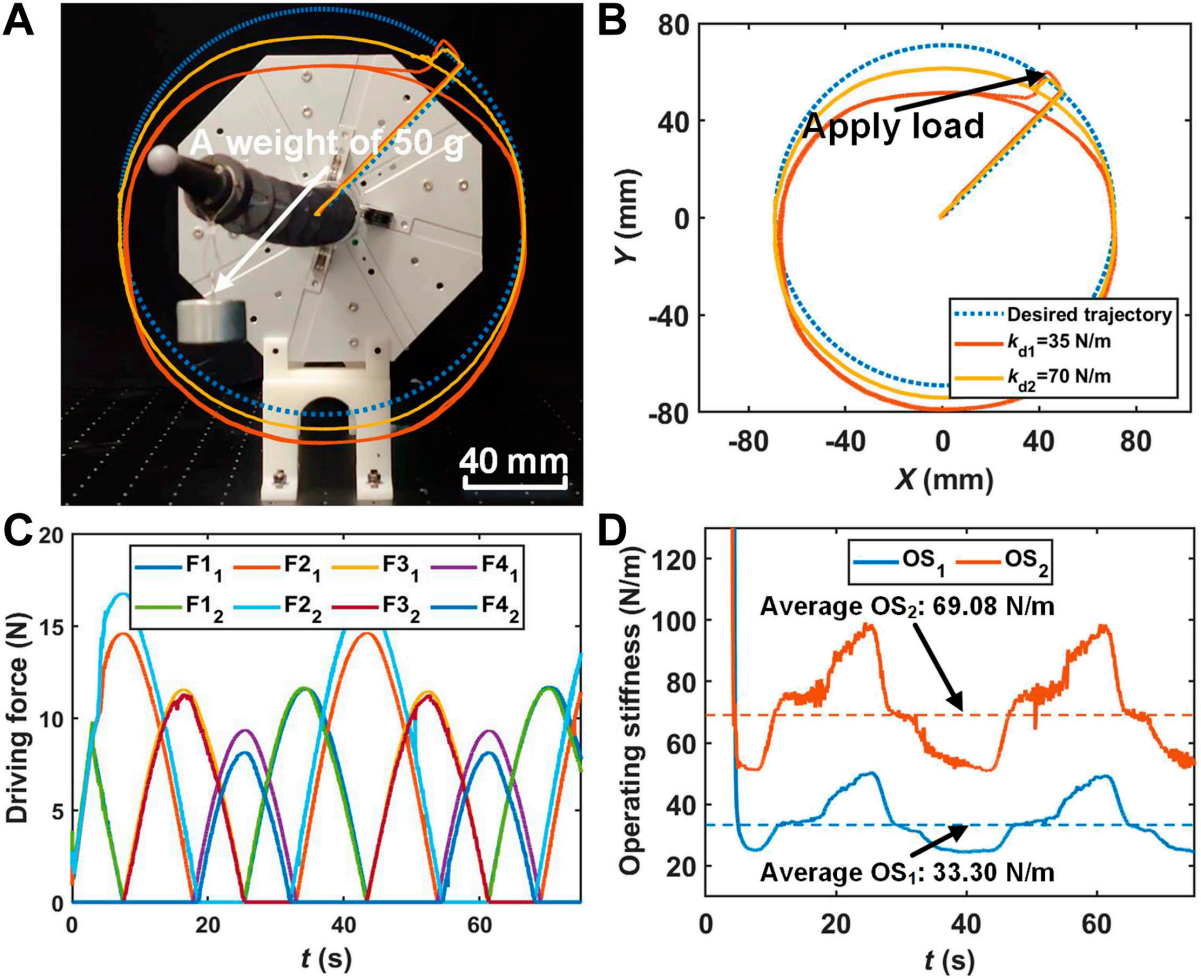
The distal tip position of the continuum manipulator and the operating stiffness curve in the operating stiffness control experiment with constant desired operating stiffness on the circle trajectory. (A) Physical picture of the operating stiffness control experiment. (B) Distal tip position of the continuum manipulator in the experiment. (C) Driving force on each driving tendon in the experiment. (D) Operating stiffness of the continuum manipulator in the experiment. Note: F*i_j_* and OS*_j_* in this figure respectively denote the driving force on the *i*th driving tendon and the operating stiffness in the *j*th experiment.

The experimental results are illustrated in Fig. 6 andTable 4. The OSC generated an average relevant error of 3.09% between the actual and desired operating stiffness. The max relevant error between the actual and desired operating stiffness produced by the proposed OSC was 41.33%, which is much larger than the average relevant error. This occurs because the configuration changes induced by the motion of the continuum manipulator result in fluctuations in the operating stiffness. For more details, please refer to Discussion. Based on the experimental results mentioned above, it can be concluded that the proposed OSC remains effective in altering the actual operating stiffness of a continuum manipulator in alignment with the desired operating stiffness, even when the continuum manipulator is in motion. Moreover, in this experiment, the continuum manipulator maintained a constant desired stiffness under load, coinciding with the operation stage in actual surgery. These experimental results also show that the proposed OSC has the potential to be efficiently applied in actual surgeries.

### Experiment of the presented OSC with time-varying desired operating stiffness at the fixed distal tip position

The following experiment was carried out to demonstrate the online adjustment of the operating stiffness of the continuum manipulator utilizing the proposed OSC. Different from the “Experiments with constant desired operating stiffness at the fixed distal tip position” section, a 50-g weight was hung at the distal tip of the continuum manipulator during this experiment, and the operating stiffness was set as a time-varying function 20 sin (*t*) + 30. The variations in the distal tip position and driving force of the continuum manipulator were observed during the experiment.

**Fig. 7.**
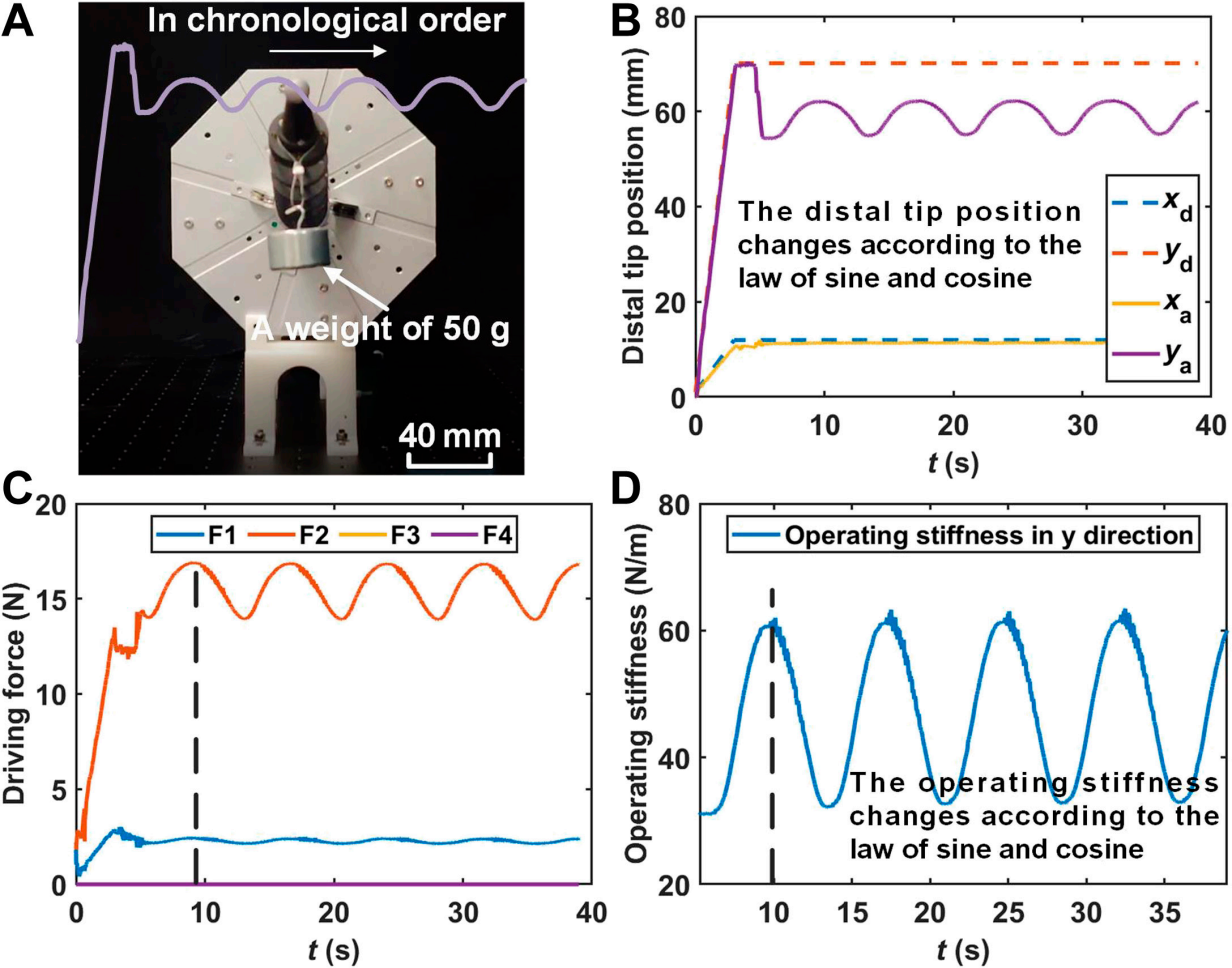
The distal tip position of the continuum manipulator and the operating stiffness curve in the operating stiffness control experiment with time-varying desired operating stiffness at the fixed distal tip position. (A) Physical picture of the operating stiffness control experiment. (B) Distal tip position of the continuum manipulator in the experiment. (C) Driving force on each driving tendon in the experiment. (D) Operating stiffness of the continuum manipulator in the experiment. Note: F1 to F4 in (C) represent the driving force on the first to fourth driving tendon, respectively.

The results of the experiments have been analyzed, as depicted in Fig. 7. In Fig. 7B, even when the load at the distal tip remains constant, the distal tip position of the continuum manipulator still fluctuates under a cosine wave pattern. This behavior is because the operating stiffness of the continuum manipulator also fluctuates under the prescribed cosine-varying function, as shown in Fig. 7D. These results substantiate that the proposed OSC can dynamically adjust the operating stiffness of the continuum manipulator in real time. Moreover, throughout the entire duration of the operating stiffness variation, the continuum robot system remains stable, demonstrating the stability of the proposed OSC. Additionally, the trend of operating stiffness corresponds to the trend in driving forces, as shown in Fig. 7C and D. Specifically, the driving force and operating stiffness reach the first peak at around 10 s and fluctuate synchronously with a period of about 7 s. This observation validates the correctness of Eqs. 5 to 7, which indicate that the operating stiffness of the continuum manipulator increases with the increase in its driving force.

## Discussion

The simulation and experimental results demonstrate that utilizing the proposed OSC to quantitatively adjust the operating stiffness of the continuum manipulator according to the desired operating stiffness is feasible and effective. Furthermore, the simulation and experimental results also indicate that, compared with the discontinuous operating stiffness adjustments arising from the nature of continuum materials and specific physical structures, the utilization of OSC allows for a more continuous and flexible modulation of the operating stiffness of the continuum manipulator. Moreover, “Experiments with constant desired operating stiffness at the fixed distal tip position” and “Experiments with constant desired operating stiffness on the circular trajectory” sections are respectively similar to the approaching and operation stages in MIS. Specifically, in the “Experiments with constant desired operating stiffness at the fixed distal tip position” section, the continuum manipulator demonstrated the ability to adjust its stiffness from low to high. This ability can meet the stiffness requirements in MIS’s approach stage. In the “Experiments with constant desired operating stiffness on the circular trajectory” section, the continuum manipulator precisely acts with greater stiffness. This action is similar to the actual action in MIS’s operation stage. These experimental results show that the designed OSC can continuously and quantitatively adjust the operating stiffness of continuum manipulators in medical surgery applications.

In the simulations and experiments with constant desired operating stiffness, quantitative calculations and comparisons of the operating stiffness of the continuum manipulator were conducted, which has been lacking in existing works. The average operating stiffness error in both simulation and experiments is below 10%, and the average standard deviation of the operating stiffness is 12.07 N/m. These minor errors and standard deviations indicate that the proposed OSC can accurately adjust and maintain the operating stiffness of the continuum manipulator. In the experiment on the circular trajectory, there was significant fluctuation in the operating stiffness of the continuum manipulator. These fluctuations arise from the relationship between the external force-induced moment and the moment generated by the continuum manipulator’s elasticity. When the continuum manipulator is in an upward-bending configuration, these two moments add up, resulting in lower operating stiffness. Conversely, when the continuum manipulator is in a downward-bending configuration, these two moments counteract each other, leading to higher operating stiffness. However, the operating stiffness of the continuum manipulator remains near the desired operating stiffness (with an average error of 3.09%).

In simulations and experiments with desired time-varying operating stiffness, a qualitative approach was employed to assess the performance of OSC. This is because a universal and effective method to quantitatively calculate the continuum manipulator’s operating stiffness in this situation does not exist. The results demonstrate that, for the desired time-varying stiffness, OSC can still successfully regulate the operating stiffness of the continuum manipulator.

## Conclusion

This work proposes a Lagrangian dynamic-based OSC for stiffness control of the continuum manipulator. The proposed OSC controller exhibits superior dynamic performance and interaction capabilities compared with static-based controllers. This controller comprises variable impedance control and feedback linearization modules. The variable impedance control module generates the distal contact force of the continuum manipulator through the distal tip motion and given impedance parameters. The Lagrangian dynamic-based feedback linearization module transforms the distal tip motion state of the continuum manipulator into the sum of the tension in the drive tendons and the contact force. By taking the difference between the outputs of these two modules, the controller outputs the required driving force without the contact force term. This result allows the OSC to control the operating stiffness without needing distal force sensors, aligning with the compact distal structure of the continuum manipulator. Additionally, the stability of the OSC is proven via an energy-weighted Lyapunov function. Moreover, this work defines the operating stiffness of the continuum manipulator. The definition is decomposed into active and passive adjustment terms based on the Lagrangian dynamics model. This result demonstrates that operating stiffness can be actively adjusted through driving forces, which is the foundation for controller design. Finally, quantitative analysis and evaluation of the controller’s performance are conducted based on the operating stiffness definition. The experimental and simulation results confirm the controller’s ability to adjust the operating stiffness of the continuum manipulator according to different desired stiffness values.

Future research will integrate the distal force sensor and the body shape sensor based on fiber Bragg grating (FBG) technology [12,13,49,50] into the continuum manipulator. Such integration supports implementing a tailored closed-loop controller and facilitates achieving more precise and stable operating stiffness control. Methods such as fuzzy control algorithms will be utilized to estimate the friction on the continuum manipulator’s driving tendons to obtain better control effects. Such quantitative analysis methods for dynamically desired operating stiffness will be proposed to evaluate the performance of the introduced OSC in a broader and more general context. Moreover, the proposed OSC will be implemented on continuum manipulators specially designed for the target medical applications [5,10,51].

## Data Availability

The data that support the findings of this study are available from the corresponding author upon reasonable request.
